# Supplementary material for: Automatic identification of compounds in molecular mixtures from liquid-phase infrared spectra
Source: Chem Sci. 2026 Jun 1;17(27):13465–78. doi: 10.1039/d6sc01583b (PMC13224293; doi:10.1039/d6sc01583b)
Supplement: SC-017-D6SC01583B-s001 [file SC-017-D6SC01583B-s001.pdf]

This journal is © The Royal Society of Chemistry 2026

Supplementary Information for: Automatic Identification of Compounds in Molecular Mixtures from Liquid-Phase Infrared Spectra

Yannah J.U. Melle,[1,2] **Thanh Nguyen,**[3] **Jeffrey Lopez,**[3,*] **Daniel Schwalbe-Koda**[1,*]

[1]Department of Materials Science and Engineering, University of California, Los Angeles, CA, USA
[2]Department of Chemistry and Biochemistry, University of California, Los Angeles, CA, USA
[3]Department of Chemical and Biological Engineering, Northwestern University, Evanston, IL, USA
[*]Correspondence to: dskoda@ucla.edu; jlopez@northwestern.edu

S1. Supplementary Text

S1.1. Gas-Phase spectra broadening and identification accuracy

Both gas- and liquid-phase pure component spectra were used as the basis set to deconvolve liquid-phase mixture spectra to identify components. When using gas-phase component spectra, the identification accuracy for two component mixtures was found to be 15.4%, compared to the 73.6% identification accuracy when using liquid-phase pure component spectra as the basis set, as shown in Fig. S1. This result indicates that gas-phase information alone is insufficient for identifying liquid-phase mixtures. Peaks in each gas-phase spectrum were broadened with a Gaussian kernel to approximate liquid-phase spectral shapes, which increased identification accuracy to 22.3%. This minimal accuracy improvement demonstrates that simple peak broadening cannot compensate for the interaction-driven spectral differences between gas-phase and liquid-phase spectra. Gaussian convolution does not significantly improve identification accuracy because it only widens gas-phase peaks. Without introducing frequency shifts, mode coupling, or intensity differences that characterize liquid-phase spectra, spectral shapes produced from broadening match neither gas-phase spectra nor true interaction-modified liquid-phase features, resulting in poor performance.

S1.2. Identification accuracy across prediction criteria and basis set size

Under the strictest evaluation criterion, which requires identification of both components from the top-2 NNLS coefficients, expanding the pure-component basis to include spectra not present in the mixtures reduces component identification accuracy (Fig. 3a). The inclusion of additional pure-component spectra expands the solution space of the NNLS decomposition. This change reduces the relative coefficient dominance of true components among the top k coefficients by introducing more candidate spectra whose spectral features partially reproduce the mixture spectral features. Expanding the pure-component spectral basis decreases spectral error because the larger set of candidate spectra gives the NNLS algorithm more flexibility to approximate the mixture by distributing coefficient weight across the additional spectra (Fig. S4). This allows coefficients associated with the true components to decrease in magnitude while permitting them to remain among the top-2. A false spectrum coefficient would overtake a true one only when the spectral shape is sufficiently close and it reduces the reconstruction error more. When the true and false molecules have nearly indistinguishable spectra, the algorithm cannot reduce the squared error by assigning more weight

to a true molecule than to a nearly identical false one, leading to an incorrect identification. This behavior explains the gradual, not sharp decline in identification accuracy when using NNLS as more pure-component spectra are included in the basis set (Fig. 3). In contrast, the interpolation method selects the single pair of spectra that yields the lowest residual, so any marginally better matching false spectrum fully replaces a true one, causing accuracy to drop more rapidly as the number of available false pure spectra increases. Evaluating identification accuracy under a less restrictive criterion where both true components are only required to be found from the top-5 coefficients increases identification accuracy from 64% to 80% with the largest basis set size. Non-true mixture components within the top-k correspond to molecules with partial similarity to true components, revealing plausible structural motifs or subcomponents that can guide interpretation and illustrate that NNLS provides meaningful coefficients. Furthermore, in settings where the number of mixture components is unknown, considering a larger k could be useful for narrowing candidates to a manageable set of chemically related structures.

S1.3. Deconvolution behavior of regularized and non-regularized linear algorithms

The coefficient distributions from regularized and non-regularized algorithms illustrated in Figure S6 show that regularization shifts coefficient values toward smaller magnitudes. For both LS and NNLS, regularization compresses the coefficient distribution toward zero, suppressing the magnitude of true component contributions. In contrast, the non-regularized solutions exhibit broader coefficient distributions. For non-regularized NNLS in particular, a clear separation emerges between very small coefficients and a smaller subset of larger coefficients, as shown in Figure S6c. This bimodal distribution structure indicates that non-regularized NNLS distinguishes components whose spectra contribute strongly to the mixture spectrum from those that contribute minimally. Regularization suppresses this separation by shrinking larger coefficients and consequently reducing the model's ability to differentiate dominant contributors. The consequence of this regularized deconvolution behavior is significantly lower identification accuracy.

The spectral MSE distributions show that non-regularized solutions achieve substantially lower spectral reconstruction error than their regularized counterparts for both LS and NNLS (Fig. S5). This is consistent with the coefficient behavior: when the coefficients are not forced toward zero, sufficient coefficient weight can be allocated to the set of dominant pure-component basis spectra, allowing the mixture spectrum to be reconstructed more accurately. By contrast, regularization limits the model's ability to represent components that share many spectral features with the mixture, which leads to increased spectral error.

The coefficient MSE values relative to the ideal molar ratios used to generate the simulated mixture spectra further support this interpretation. In the MD simulations, each mixture was constructed from equal molar amounts. Thus, for a two-component mixture, an ideal mixture model would assign coefficients of 0.5 to the two true components, and zero valued coefficients to all other components. However, the recovery of exactly 0.5/0.5 coefficients is not expected from a linear deconvolution model because the liquid-phase spectra arise from intermolecular interactions and nonlinear mixing effects whose spectra are not linear combinations of the pure-component spectra.

Within the linear approximation, non-regularized NNLS exhibits the smallest median and mean coefficient error relative to the ideal 0.5 reference value, indicating that the true components' coefficients are closest to the underlying molar composition (Table S1). Conversely, non-regularized LS shows larger deviations from ideal mixing. Least squares can assign negative coefficients to certain basis spectra to reduce the mixture spectrum reconstruction error. However, these negative coeffi-

cients are not physically meaningful, even when the magnitude is used to evaluate deviation from ideal molar compositions. As a result, LS produces coefficients with larger ideal coefficient MSE than NNLS, suggesting that enforcing non-negativity constrains the resulting coefficient solution toward physically meaningful mixing weights. The MSE between the ideal and NNLS coefficient values increases over basis set size because candidate molecules whose spectral features can additionally represent the mixture spectrum are allocated coefficient weight, thus reducing the true component coefficient values further from the ideal value of 0.5.

Table S2 shows that the deconvolution algorithms produce less sparse (more non-zero) coefficients as the pure-component dataset size increases. Notably, NNLS produces sparser coefficient vectors than LS and both of the regularized algorithms, further indicating that using NNLS results in the true component coefficient values deviating less from the ideal coefficient values as more component spectra that can approximate the mixture spectrum are added.

Table S1: Mean squared error summary statistics of the coefficients obtained by solving regularized and non-regularized algorithms versus the ideal coefficients (0.5 for each of the true components).

algorithm	basis set size	median	mean	std	n mixtures
LS	328	4.39×10^{-2}	5.23×10^{-2}	4.01×10^{-2}	27657
LS REG	328	2.47×10^{-1}	2.47×10^{-1}	1.05×10^{-3}	27657
NNLS	328	4.60×10^{-2}	5.73×10^{-2}	4.43×10^{-2}	27657
NNLS REG	328	2.47×10^{-1}	2.47×10^{-1}	1.05×10^{-3}	27657
LS	1640	1.44×10^{-1}	1.50×10^{-1}	2.94×10^{-1}	27657
LS REG	1640	2.48×10^{-1}	2.48×10^{-1}	8.98×10^{-4}	27657
NNLS	1640	5.68×10^{-2}	6.75×10^{-2}	4.79×10^{-2}	27657
NNLS REG	1640	2.48×10^{-1}	2.48×10^{-1}	9.02×10^{-4}	27657
LS	3280	1.82×10^{-1}	1.77×10^{-1}	4.55×10^{-2}	27657
LS REG	3280	2.48×10^{-1}	2.48×10^{-1}	8.40×10^{-4}	27657
NNLS	3280	6.33×10^{-2}	7.34×10^{-2}	5.04×10^{-2}	27657
NNLS REG	3280	2.48×10^{-1}	2.48×10^{-1}	8.51×10^{-4}	27657
NNLS	4920	6.55×10^{-2}	7.47×10^{-2}	4.98×10^{-2}	27645
NNLS	8528	7.24×10^{-2}	8.06×10^{-2}	5.14×10^{-2}	27635

S1.4. Sequential addition and removal metrics for component contribution analysis

Quantifying spectral metrics (as described in 5.5) in the presence or absence of specific components allows analyzing multi-component mixtures in terms of relative contributions to predict the component count. Cases in which a component shows a large NNLS coefficient but a small or even negative incremental contribution indicate that its spectral features are redundant with those of other components. Sequential-removal metrics make this redundancy explicit by removing a component and recomputing coefficients to reveal whether other spectra can substitute for its features. If the reconstruction improves upon the removal, the removed component was not uniquely informative and could have had broad or overlapping features that can be captured by other spectra that simultaneously capture more of the mixture spectrum. Conversely, if the reconstruction worsens, no other spectra can compensate for the features it describes in the mixture spectrum, indicating that it provides unique information about the mixture.

Table S2: Average number of non-zero coefficients per mixture for each algorithm and basis set size, where coefficients are considered non-zero if greater than 10^{-6}.

algorithm	basis set size	mean non-zero count
LS	328	166
LS	1640	824
LS	3280	1645
LS REG	328	324
LS REG	1640	1432
LS REG	3280	2491
NNLS	328	19
NNLS	1640	28
NNLS	3280	30
NNLS	4920	33
NNLS	8528	44
NNLS REG	328	324
NNLS REG	1640	1388
NNLS REG	3280	2222

This coefficient redistribution behavior also offers a way to reason about molecules missing from the basis set that may be present in the mixture. When a dominant but incorrect predicted component is removed and the mixture reconstruction improves, the components assigned larger coefficients in the re-solved model approximate the structure of the true but absent molecule more closely than the removed one does, providing structural guidance about unknown mixture components. When components are removed from the basis set independently, those that are ranked higher by their solved NNLS coefficient contribute to a greater reconstruction error compared to the true mixture spectrum, shown in S7a. This result is further evidence that NNLS coefficients quantify the importance of the basis component spectra to describe the mixture spectra. When the true components of mixtures are removed from the basis set and the NNLS coefficients are recomputed in the absence of the true components, average reconstruction error is also larger than the average reconstruction error of mixtures deconvoluted with their true components in the basis set (Fig. S7b). The average reconstruction error observed when true components are present in the basis set provides a baseline for the quality of fit achievable when the correct molecules are available in the pure component dataset used to deconvolute mixtures. Comparing the reconstruction error of a given mixture to this baseline can indicate that the mixture cannot be sufficiently described by the available basis set components, suggesting that one or more true components may be absent from the database.

S1.5. Spectral similarity patterns in false component predictions

Among the misidentified mixtures, the cumulative intensity differences clarify how the NNLS and interpolation component predictions differ in the spectral characteristics that lead to misclassifications. For each mixture, average cumulative distribution function (CDF) differences were computed between the two true component spectra (true-true pairs), between true and falsely predicted components (false-true pairs), and between two falsely predicted components (false-false pairs), shown in Figure S8a. This analysis was done for all mixtures misidentified across all basis-set sizes.

For both the false-true and false-false pair types, the NNLS distributions are shifted toward smaller spectral differences compared to interpolation. Both NNLS and the interpolation method produce false-true pairs that are more spectrally similar than random pairs and are also more similar than the true-true pairs. Further, the true-true spectral differences are comparable to or larger than the random pairings. These two results suggest that misclassifications do not arise because the true components in a mixture are inherently too similar but rather due to candidate components being closer in spectral space than typical true pairs.

Importantly, NNLS false-true pairs have smaller average and median CDF differences than the interpolation false-true pairs, indicating that when NNLS selects a false component, it is more spectrally similar to the true component than a false component selected by the interpolation method is. The same pattern for false-false pairs is seen as well: NNLS false-false pairs are spectrally closer than false-false pairs from interpolation (and random pairings). NNLS failures tend to select two false components that are close to one another in spectral space, whereas the interpolation method's two false components resemble random pairings. This behavior is consistent with the NNLS algorithm that optimizes continuous coefficients over the entire pure-component basis set. Near-neighbor spectra can receive partial coefficient weighting, resulting in the spectrum of a falsely identified molecule lying close to the correct molecule's spectrum in spectral space. Conversely, interpolation evaluates discrete candidate pairs, which leads to incorrect selections that are more spectrally distinct.

On the mixture level, reconstructed spectra for the misidentified mixtures were generated using the coefficients obtained from the NNLS and interpolation methods. Mixtures reconstructed with NNLS coefficients are more spectrally similar to the true mixture spectra than those produced using interpolation coefficients (Fig. S8 b). This result is consistent with the pure-component pair analysis. By design, NNLS retains flexibility to approximate the true mixture spectrum even when the identified components are incorrect because the algorithm optimizes continuous coefficients over the full basis set. In contrast, the interpolation method is restricted to discrete candidate pairs and is thus limited in reproducing the mixture spectrum, resulting in larger spectral deviations when misidentifications occur.

S2. Supplementary Methods

These Supplementary Methods describe in more detail the additional results and calculations in the Supplementary Information.

S2.1. Gaussian broadening of gas-phase spectral peaks

Gaussian broadening was applied to simulated gas-phase spectra by convolving the spectra with a normalized Gaussian kernel. The kernel was constructed over a window of fixed size, centered at zero, and its width was controlled by the standard deviation parameter, which determines the spread of the Gaussian. The Gaussian kernel standard deviation was 20 cm^{-1} (corresponding to $\sigma = 5$ grid points on a spectral grid with 4 cm^{-1} spacing), yielding a full width at half maximum (FWHM) of 47.1cm^{-1}. The kernel was normalized to ensure spectral intensity values were preserved after the convolution was applied, and the output spectrum retained the same length as the original input spectrum. This procedure smooths sharp spectral features by redistributing intensity locally across neighboring wavenumbers. It was used to approximate the peak broadening that is observed between gas-phase and liquid-phase spectra.

S2.2. Shifting peaks method

The peak shifting analysis reported in Fig. 2c was performed using mixture spectra constructed as linearly weighted sums of pure-component spectra. Each pure spectrum was modified using a local peak-shifting procedure. Randomly selected spectral windows were shifted by amounts sampled from a normal distribution, resulting in local displacements in peak positions while preserving the overall spectral structure. A smooth blending procedure was applied when inserting the shifted windows to avoid discontinuities in the spectrum, and the spectrum was rescaled to preserve total intensity after shifting. The same shifts were applied consistently to each molecule's pure spectrum to preserve its spectral identity. The NNLS algorithm was then applied to obtain coefficients for the linearly combined mixtures using the shifted pure spectra basis set.

S2.3. Atom match filtering method

Atom count information for molecules in each mixture was used to filter implausible pure-component predictions obtained using NNLS coefficients (Fig. 3b). For a given mixture, the total number of each atom type was enumerated from the true mixture composition. In practice, this elemental composition information could be obtained using mass spectroscopy without knowledge of the specific components present in the mixture.

Assuming an n-component mixture (here, $n = 2$), all pure-component combinations whose summed atom counts exactly matched the mixture atom counts were identified. The set of molecules appearing in any valid combination was taken as the plausible candidate pool for predicting that mixture's components. The NNLS algorithm was used to obtain coefficients for all pure components, as described in section 5.3. The coefficients were then restricted to those corresponding to molecules in the plausible candidate pool, and the top k candidates ranked by absolute coefficient magnitude were used to evaluate identification accuracy as described in section 5.4.2.

S2.4. Fragment-dependent contributions to gas-liquid spectral differences and mode decomposition

Each molecule was decomposed into its Murko scaffold "core" and the largest remaining fragment. Within each core, the average CDF difference value of all molecules sharing that core was standardized (z-scored). This per-core standardization removes core-specific effects, so the resulting z-scores reflect fragment-dependent contributions to the gas-liquid spectral difference. The composition of co-fragments in the two modes for molecules with a carboxylic acid as the largest fragment was analyzed as an example in this work. Molecules with this fragment were partitioned into two modes by fitting a two-component Gaussian mixture to their per-core z-scored cumulative intensity differences (the molecule's average CDF difference minus the core-specific mean, divided by the core-specific standard deviation). Co-fragment occurrences were counted across molecules in the two modes, and the most common co-fragments were used to calculate relative compositions in each mode.

S3. Supplementary Figures

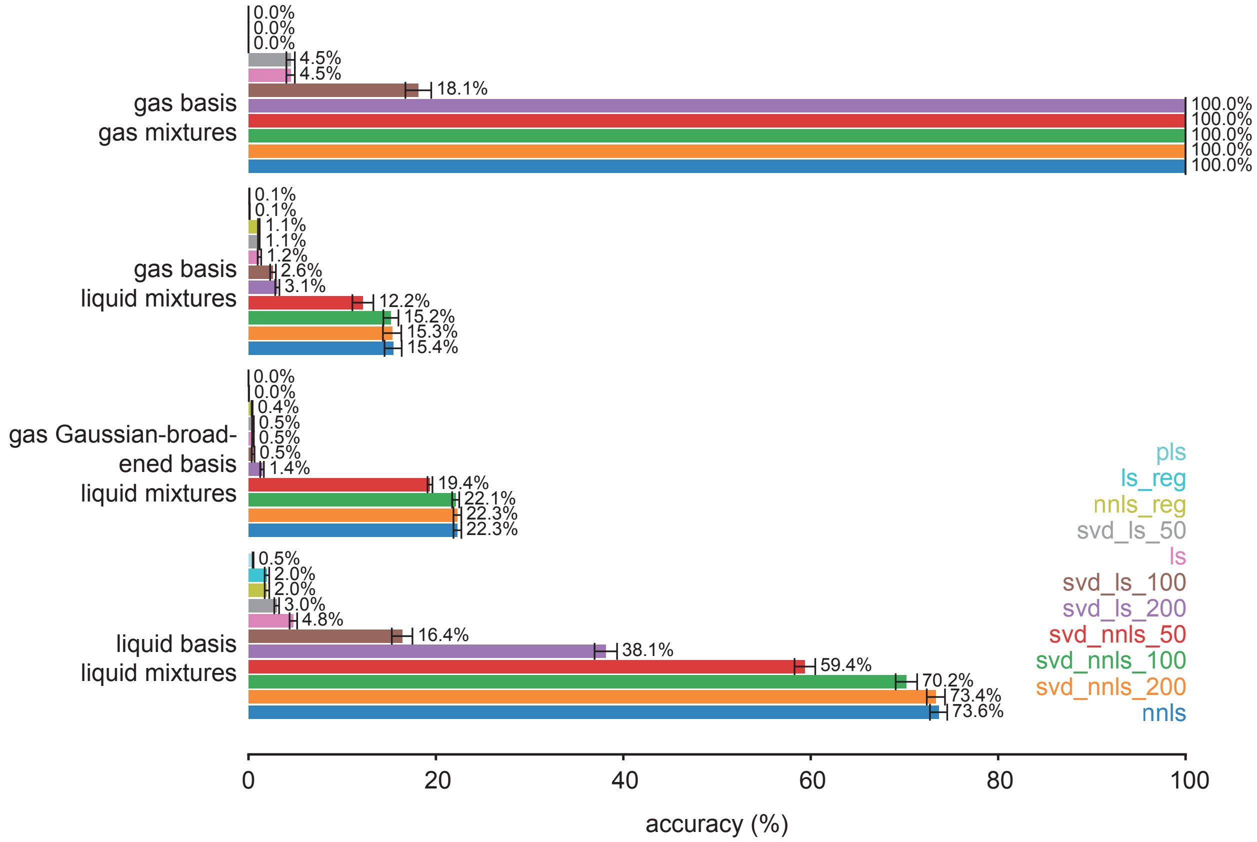

Fig. S1: Identification accuracies of unknown components of two-component liquid-phase mixtures from simulated IR spectra, using MD-generated simulated pure-component IR spectra, as described in the main text, Section 5.4.1. Algorithm labels follow the format *alg_n* where *alg* denotes the coefficient estimation method used to solve Y=CX, and n (when present) indicates the dimensionality of the singular value decomposition (SVD) latent space onto which X and Y were projected prior to coefficient estimation. Methods without a numeric suffix operate in the original feature space.

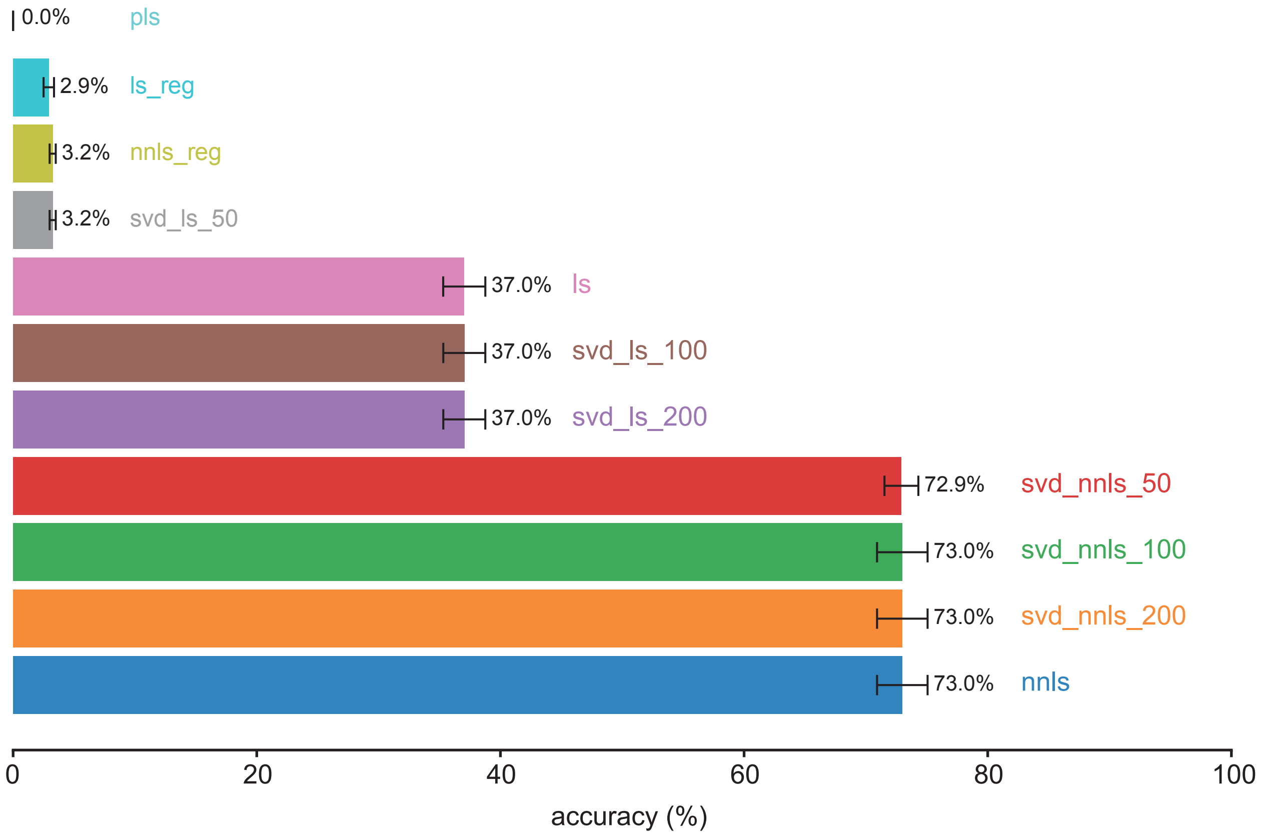

Fig. S2: Identification accuracies of unknown components of three-component liquid-phase mixtures from simulated IR spectra, using MD-generated simulated pure-component IR spectra. Algorithm labels follow the format *alg*_*n* where *alg* denotes the coefficient estimation method used to solve $Y = CX$, and n (when present) indicates the dimensionality of the singular value decomposition (SVD) latent space onto which X and Y were projected prior to coefficient estimation. Methods without a numeric suffix operate in the original feature space.

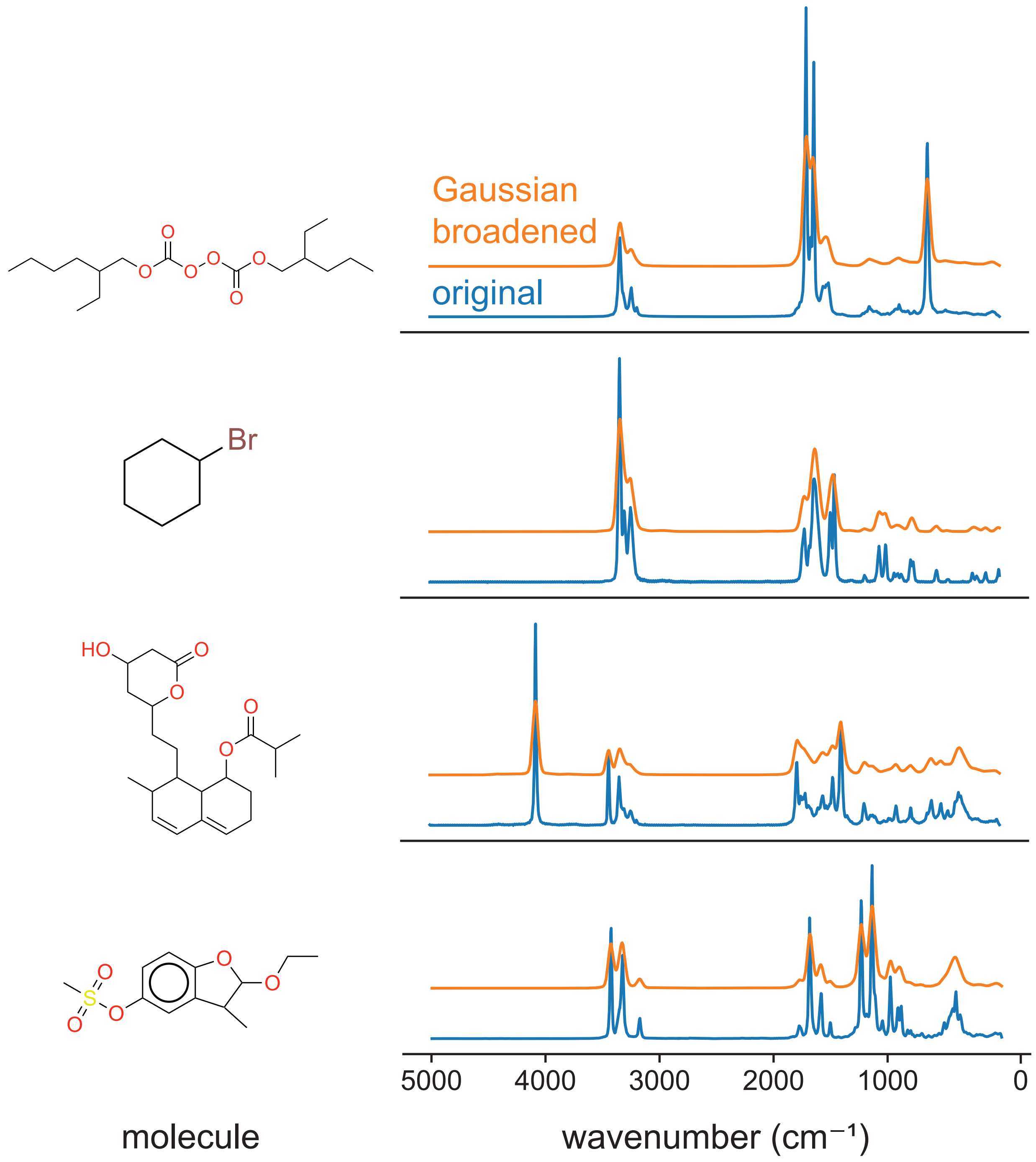

Fig. S3: Examples of simulated gas-phase spectra (blue) with their corresponding Gaussian-broadened spectra (orange), as described in the Supplementary Methods S2.

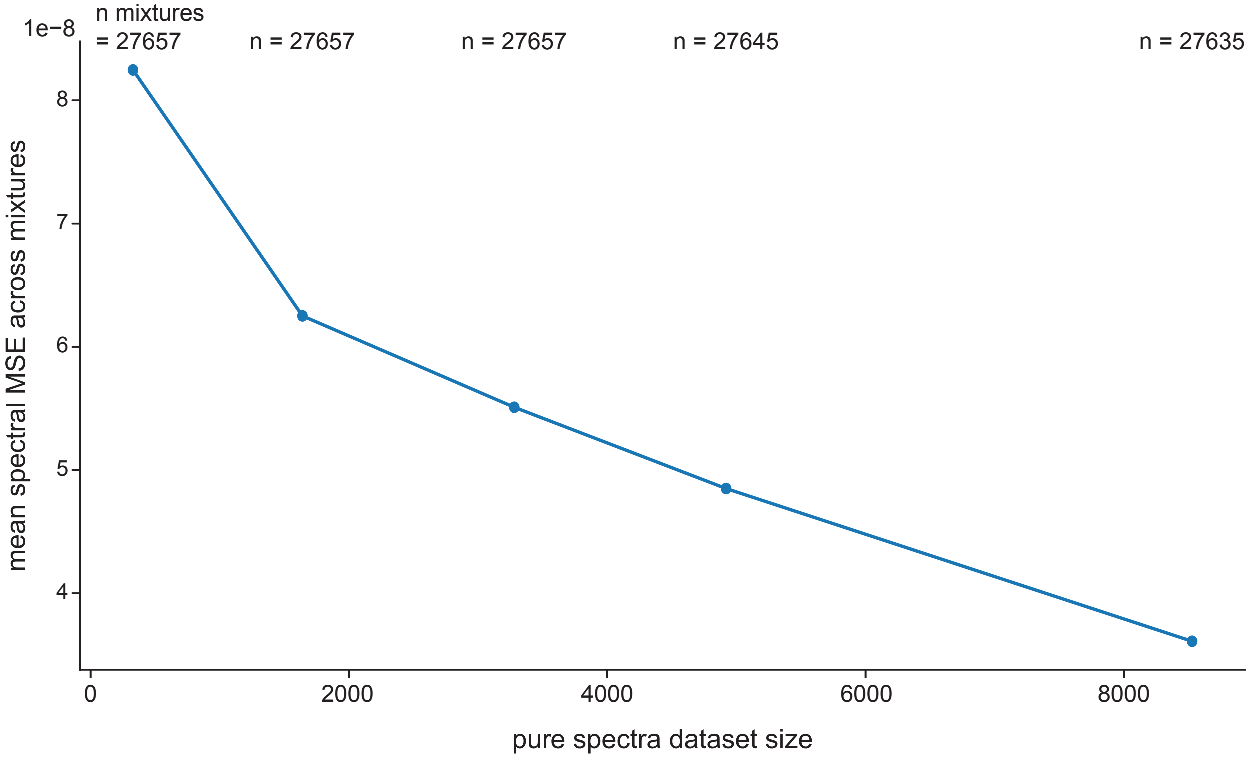

Fig. S4: Mean spectral mean squared error (MSE) and mean average cumulative distribution function (CDF) difference across all mixtures for varying pure-component basis set sizes. The number of mixtures, n, successfully deconvolved for each basis set size is indicated.

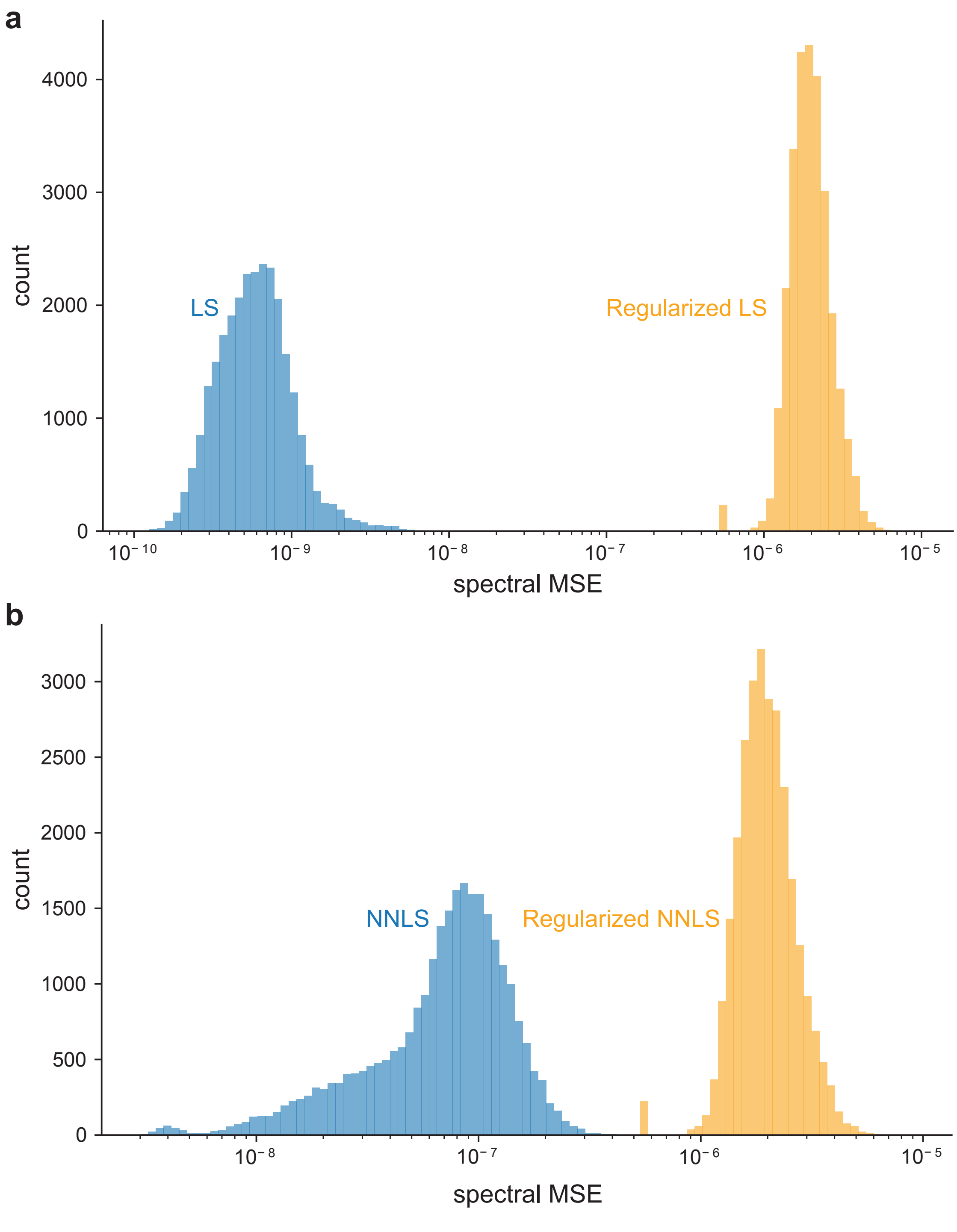

Fig. S5: Distributions of the mean squared errors (MSEs) between the true mixture spectrum and the spectrum reconstructed by each indicated algorithm, for all mixtures that were successfully deconvolved in this study across all basis set sizes. **a**, Spectral MSE distributions for the LS and Regularized LS methods. **b**, Spectral MSE distributions for the NNLS and Regularized NNLS methods.

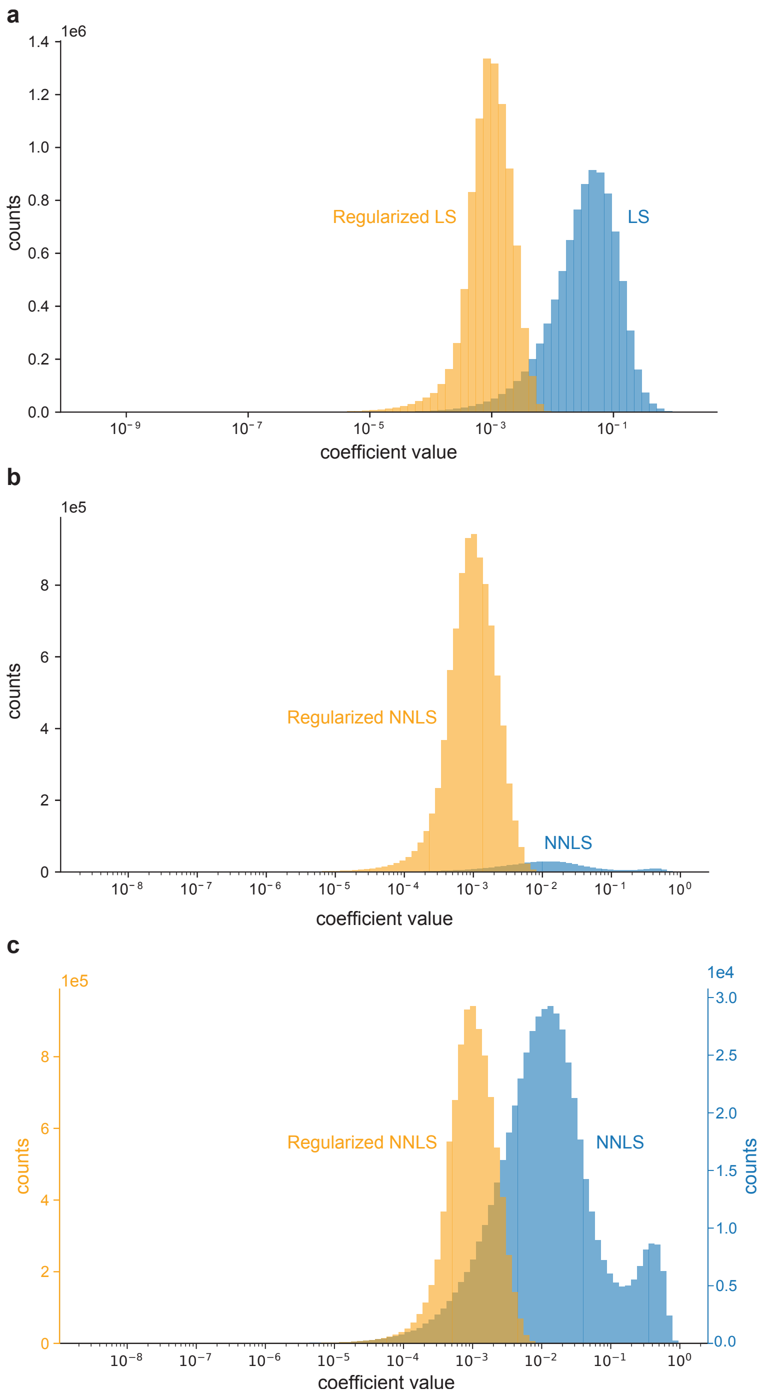

Fig. S6: Distributions of the non-zero coefficient values obtained by each indicated algorithm for all mixtures successfully deconvolved in this study across all basis set sizes. **a**, Coefficient distributions for the LS and Regularized LS methods. **b**, Coefficient distributions for the NNLS and Regularized NNLS methods. **c**, NNLS and Regularized NNLS coefficient distributions using separate y-axes for each method to better visualize the shape of the NNLS coefficient distribution.

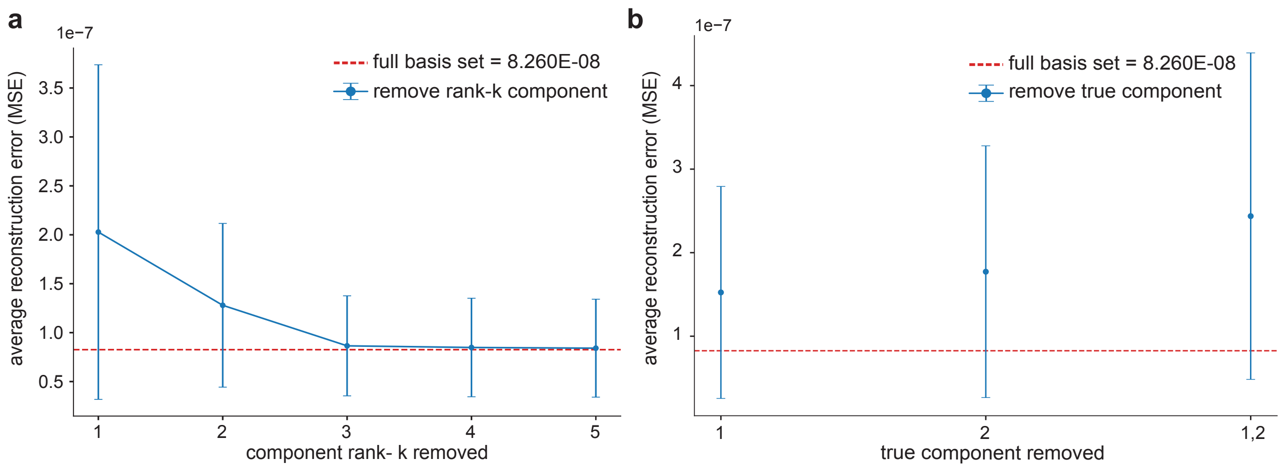

Fig. S7: Average mixture reconstruction errors (MSE) after solving for NNLS coefficients with a reduced basis for 10,000 mixtures, starting from the full minimum basis set size (328). Each rank-k component (ranked by the absolute value of the original NNLS coefficients) was separately removed from the basis set, and each true pure component and both pure components was separately removed from the basis set. Then, NNLS was used to resolve for component coefficients from the reduced basis set, and reconstruct the mixture spectrum. This reconstruction was compared with the true mixture spectrum for each mixture. **a**, Average reconstruction error after removing the rank-k basis component. Reconstruction improves with each ranked component removed, quantifying component importance by error relative to the full basis baseline. **b**, Average reconstruction error after removing each true component, and removing both true components from the basis set, all separately.

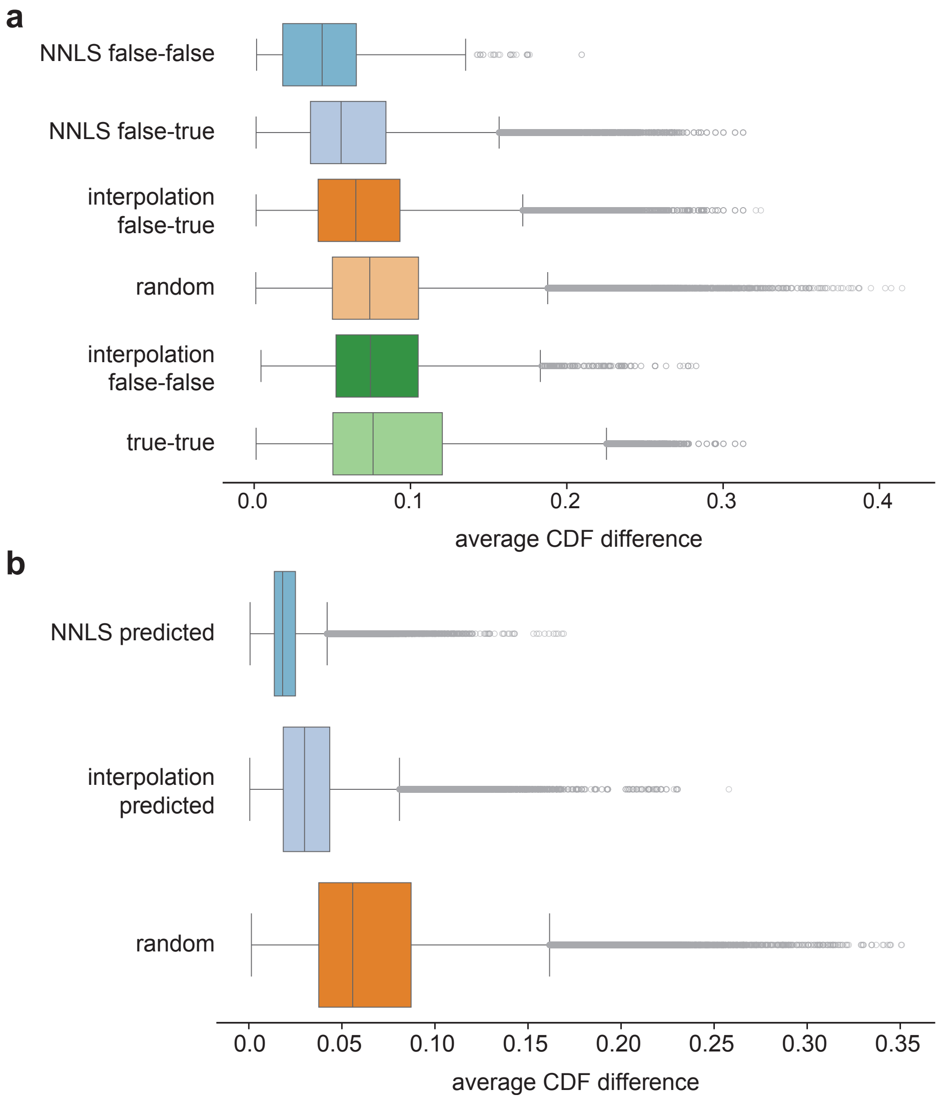

Fig. S8: Distributions of spectral differences for misidentified two-component mixtures across all basis-set sizes using the NNLS and interpolation methods. **a**, Average cumulative distribution function (CDF) differences between pure-component spectra. "True-true" denotes the two correct components in a mixture; "false-true" denotes pairs consisting of one true and one falsely predicted component; and "false-false" denotes pairs of two falsely predicted components. Random pairings (200,000 pairs) are shown for reference. NNLS false selections are shifted toward smaller spectral differences relative to interpolation, indicating that NNLS misidentifications involve components that are more spectrally similar to the true molecules. **b**, Average CDF differences between reconstructed and true mixture spectra using the NNLS and interpolation methods. Mixtures reconstructed by NNLS are more spectrally similar to the ground truth than those reconstructed by interpolation, while random mixtures exhibit larger spectral differences (shown for reference).

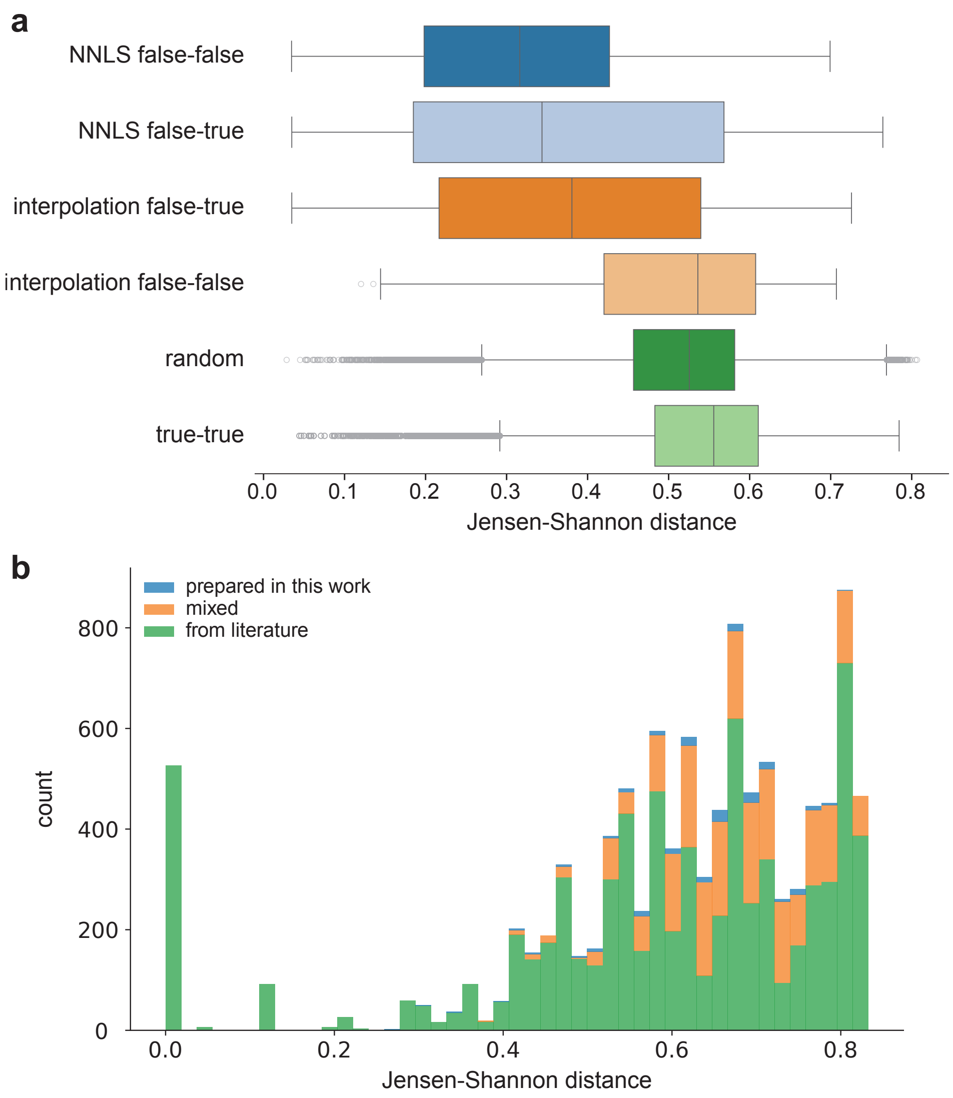

Fig. S9: Distributions of spectral differences for misidentified two-component mixtures across all basis-set sizes using the NNLS algorithm and for experimentally measured pure component spectra. **a**, Jensen-Shannon distances between pure-component spectra. "True-true" denotes the two correct components in a mixture; "false-true" denotes pairs consisting of one true and one falsely predicted component; and "false-false" denotes pairs of two falsely predicted components. Random pairings (200,000 pairs) are shown for reference. **b**, Jensen-Shannon distances between all experimentally measured pure component spectra used in the basis set to predict the components of the experimentally observed mixture spectra prepared in this work.

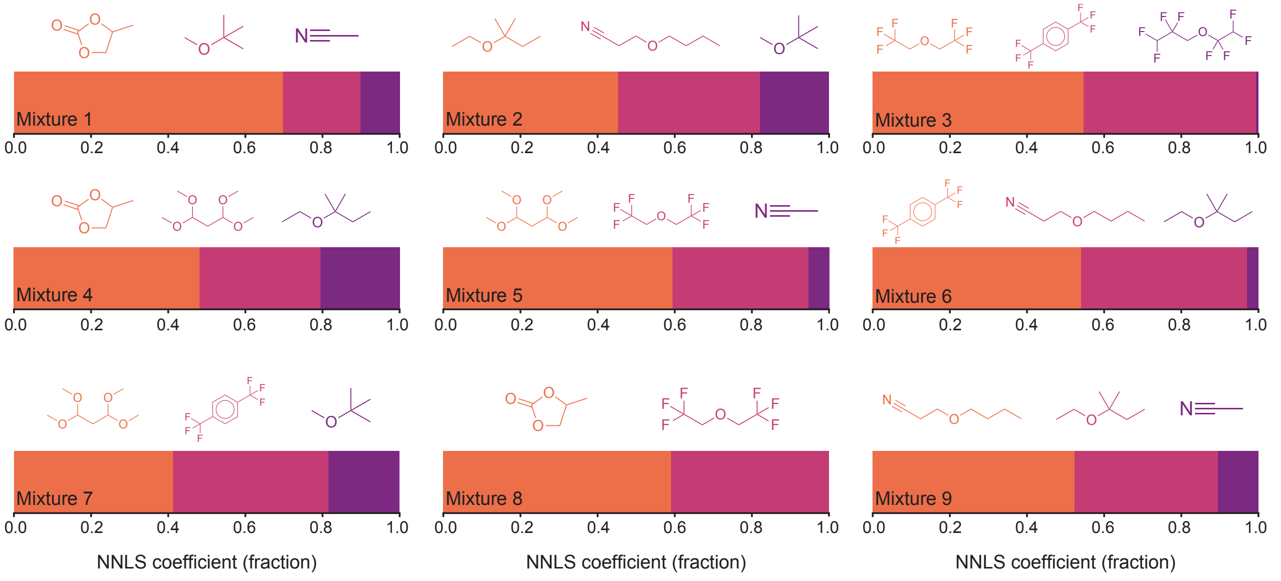

Fig. S10: Top-2 and top-3 mixture components ranked by NNLS coefficient for all experimental mixtures, as described in the main text sections 2.6 and 5.4.1.

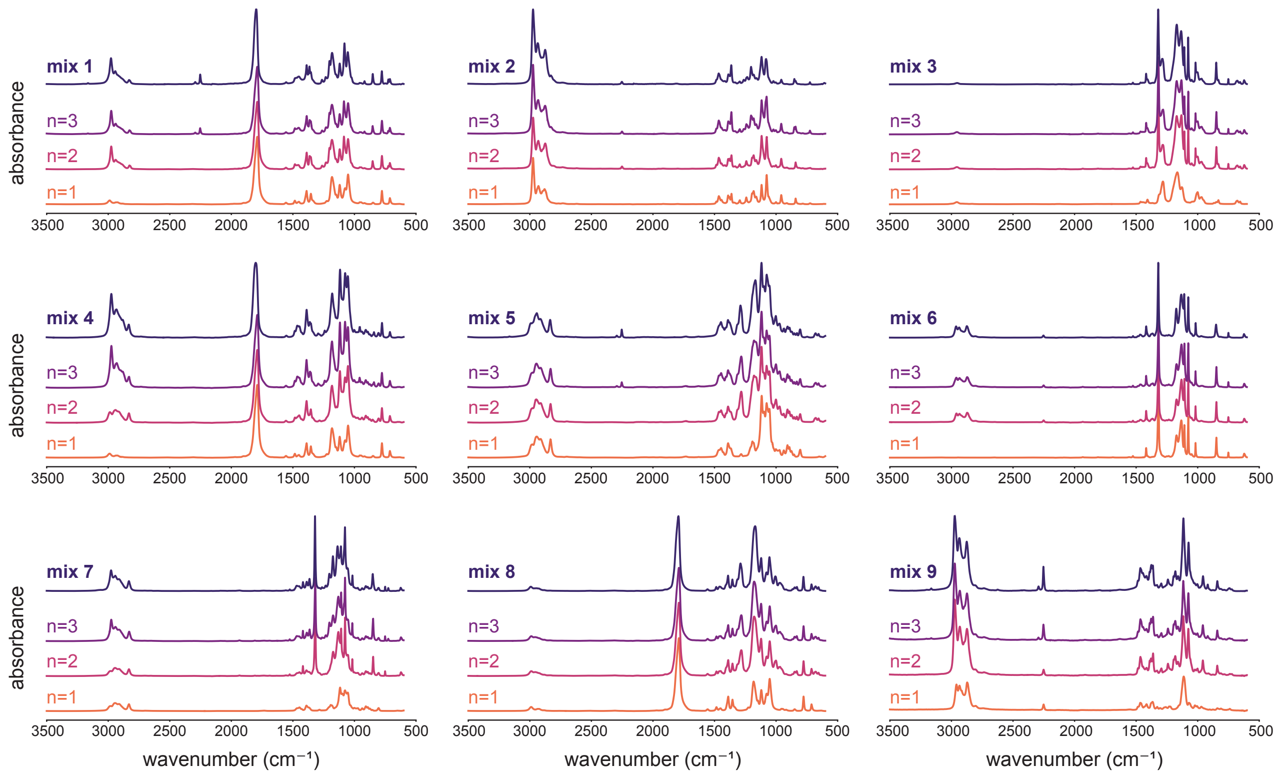

Fig. S11: Cumulative weighted spectrum reconstructions for all experimental mixtures, obtained by sequentially adding the top $n = 1 - 3$ NNLS-ranked component spectra, each weighted by its NNLS coefficient.
